# Preoperative Serum Carbohydrate Antigen 19-9 Levels Cannot Predict the Surgical Resectability of Pancreatic Cancer: A Meta-Analysis

**DOI:** 10.3389/pore.2022.1610266

**Published:** 2022-05-10

**Authors:** Márton Benke, Nelli Farkas, Péter Hegyi, Benedek Tinusz, Patrícia Sarlós, Bálint Erőss, Kata Szemes, Nóra Vörhendi, Zsolt Szakács, Ákos Szücs

**Affiliations:** ^1^ First Department of Surgery, Semmelweis University, Budapest, Hungary; ^2^ Institute for Translational Medicine, Medical School, University of Pécs, Pécs, Hungary; ^3^ Szentágothai Research Centre, University of Pécs, Pécs, Hungary; ^4^ Institute of Bioanalysis, Medical School, University of Pécs, Pécs, Hungary; ^5^ Division of Gastroenterology, First Department of Medicine, Medical School, University of Pécs, Pécs, Hungary; ^6^ First Department of Medicine, University of Szeged, Szeged, Hungary; ^7^ Clinical Medicine Doctoral School, University of Szeged, Szeged, Hungary

**Keywords:** biomarker, prognosis, pancreas adenocarcinoma, carbohydrate antigen 19-9, pancreatic surgery

## Abstract

**Background and Aims:** Pancreatic ductal adenocarcinoma has one of the worst prognosis of all malignancies. This investigated the relationship between the preoperative serum carbohydrate antigen 19-9 and surgical resectability.

**Methods:** A systematic search was performed in three databases (MEDLINE, EMBASE, and Web of Science) to compare the surgical resectability of pancreatic ductal adenocarcinoma in patients with high and low preoperative serum carbohydrate antigen 19-9 values. The receiving operating characteristic curves were constructed and the weighted mean differences for preoperative serum carbohydrate antigen 19-9 levels of resectable and unresectable groups of patients were calculated. The PROSPERO registration number is CRD42019132522.

**Results:** Results showed that there was a significant difference in resectability between the low and high carbohydrate antigen 19-9 groups. Six out of the eight studies utilised receiver operating characteristic curves in order to find the cut-off preoperative carbohydrate antigen 19-9 levels marking unresectability. The overall result from the pooled area under curve values from the receiver operating characteristic curves was 0.794 (CI: 0.694–0.893), showing that the preoperative carbohydrate antigen 19-9 level is a “fair” marker of resectability. The result of the pooled weighted mean differences was 964 U/ml (*p* < 0.001) showing that there is a significant carbohydrate antigen 19-9 difference between the resectable and unresectable groups. Based on the results of the I-squared test, the result was 87.4%, accounting for “considerable” heterogeneity within the population.

**Conclusion:** Carbohydrate antigen 19-9 is not a reliable marker of unresectability, it should not be used on its own in surgical decision-making.

**Systematic Review Registration**: clinicaltrials.gov, identifier CRD42019132522

## Introduction

Pancreatic ductal adenocarcinoma (PDAC) makes up 90% of all pancreatic malignancies but has the worst prognosis [1]. Despite all efforts, the 5-year survival rate remains below 7% [2]. The only curative treatment is surgical resection. Unfortunately, even in high-volume centers fewer than 20% of patients have resectable primary disease. More than 20% of patients who were considered operable initially, are deemed unresectable during surgery due to distant metastases or the local progression of the disease [2, 3]. There are many aspects of how to assess resectability, but at present, most decisions are made based on the results of different imaging modalities, such as computed tomography (CT), positron emission computed tomography (PET-CT), endoscopic ultrasound (EUS), and magnetic resonance imaging (MRI) adhering to the international guidelines for the criteria of resectability [4]. Serum CA 19-9 is mostly used for controlling the progression or recurrence of the disease after surgery [2]. It is known that the gradually increasing serum CA 19-9, which is the most widely-used pancreatic cancer biomarker in clinical practice, is somewhat parallel with the tumor progression, however, there is no known cut-off value that would reliably indicate unresectability. Also, high preoperative CA 19-9 levels tend to be used as a marker of unresectability although there is no evidence of its usefulness alone. The surgical procedure used to remove PDAC is a very complex and difficult procedure with high complication rates, postoperative morbidity and mortality [5]. It would be essential to decrease the number of patients who’s unresectable PDAC is discovered during surgery. On the other hand, we have to avoid any impact suggesting unresectability based on only one measurable parameter and ignoring more strong parameters resulting regrettable contraindication for surgery in a potentially resectable pancreatic ductal adenocarcinoma. The aim of this study is to objectively evaluate first this question through a systematic review and meta-analysis.

## Materials and Methods

This meta-analysis was reported in accordance with the Preferred Reporting Items for Systematic Reviews and Meta-Analyses (PRISMA) Statement [6]. The protocol was registered into the International Prospective Register of Systematic Reviews (PROSPERO) under CRD42019132522.

### Search and Eligibility

For the purposes of this research, the authors searched for publications that discussed PDAC patients who had undergone surgery with curative intent, and which also investigated the prognostic role of CA 19-9 levels for surgical resectability.

A systematic search was performed in MEDLINE (*via* PubMed), EMBASE and Web of Science from its inception until 26 April 2019. No restrictions were imposed on the search.

The search was performed with the following query:

(pancreas OR pancreas*) AND (cancer* OR tumor* OR tumour* OR neoplasm* OR malignant* OR carcinoma* OR adenocarcinoma*) AND (“CA 19-9” OR “CA19-9” OR “carbohydrate antigen 19-9” OR “cancer associated antigen 19-9” OR “cancer antigen 19-9” OR “cancer associated glycolipid antigen 19-9” OR “sialylated Lewis antigen”).

### Selection and Eligibility

The inclusion criteria for this paper was the following: only patients with radio morphologically and, in most of the cases, histologically confirmed PDAC were included; these patients were going through surgery with curative intent and had available preoperative serum CA 19-9 levels. Only those patients were included who haven’t received neoadjuvant chemotherapy. Only full texts were eligible for the analysis.

Exclusion criteria: Review articles, case reports, and articles only published in abstract form were excluded. Studies where patients had a radiologically inoperable disease, patients who received neoadjuvant chemotherapy, patients who were going through surgery or chemotherapy with palliative intent were also excluded.

After the database search, the results were imported into the EndNote X8 reference manager software (Clarivate Analytics, Philadelphia, PA, United States). Duplicates were removed. The remaining records were first screened by title, then by abstract, and finally by full text, by two authors (MB and ÁSz).

### Data Extraction

Data was extracted using a pre-defined data extraction form containing the author, year of publication, country, demographic data, study characteristics, sample size, number of patients with resectable and unresectable disease, preoperative serum CA 19-9 values, area under the receiving operating characteristics curve (AUC ROC) values, confidence intervals, sensitivity, specificity, positive predictive value (PPV), negative predictive value (NPV), true positives (TP), false positives (FP), true negatives (TN), and false negatives (FN) (Supplementary Table S1A-C). The raw data (TP, FP, TN, FN) was provided in five out of the eight studies included. In the case of two studies, these values were calculated from the known variables; in one study, there was insufficient information available to calculate them.

### Risk of Bias Assessment

Risk of bias (ROB) assessment was performed by two investigators (MB and ÁSz). To assess the ROB, the Prediction Model Risk of Bias Assessment Tool (PROBAST), as a standard tool recommended in prognostic meta-analyses, was used. This was done separately for each study by both authors; disagreements were resolved by consensus. The ROB and applicability can be assessed using PROBAST in a transparent and thorough way in studies that develop (also validate or update) prediction models for the target predictions. The PROBAST tool examines the studies based on four main domains: participants, predictors, outcome and analysis. These domains contain signaling questions to assess the possible sources of bias within the included studies. The possible answers to these signaling questions are “yes,” “no,” or “unclear” in order to assess the ROB and the applicability of the individual studies. The domain was deemed to have a high overall ROB if at least one of the signaling questions was answered “no” or “probably no.” The domain was overall “unclear” if the answer to at least one of the signaling questions was “unclear.” Overall, a low ROB was only possible if all signaling questions were answered “yes” or “probably yes.”

### Statistical Analysis

The meta-analytical calculations were performed using STATA version 15.0 (Stata, College Station, TX, United States). The DerSimonian and Laird random-effects models were applied to calculate the pooled estimate effect sizes across the studies.

The AUC values and their confidence intervals were collected to examine the prognostic ability of the CA 19-9 marker for surgical resectability. The classification of AUC results based on the Cochrane handbook are the following: 90–100 = excellent; 80–90 = good; 70–80 = fair; 60–70 = poor; 50–60 = fail [7]. Pooled sensitivity and specificity were also calculated from the raw data (TP, TN, FP, FN) given in the studies. Additionally, weighted mean differences (WMDs) were calculated to detect the differences between the CA 19-9 levels of resectable and unresectable cases. The heterogeneity was tested using Cochran’s Q (χ^2^) and I^2^ statistic. I^2^ represents the percentage of the total variability, which cannot be explained by random chance. I^2^ values under 40% show that heterogeneity might not be important, 30%–60% represents moderate, 50%–90% substantial, and above 75% represents considerable heterogeneity. An SROC analysis was also planned as part of this research; however, the requirements for it were not met described in the paper written by Catherine M. Jones et al. [8] Publication bias was assessed using a visual inspection of funnel plots, due to the low number of publications.

## Results

### Search Results

**FIGURE 1:**
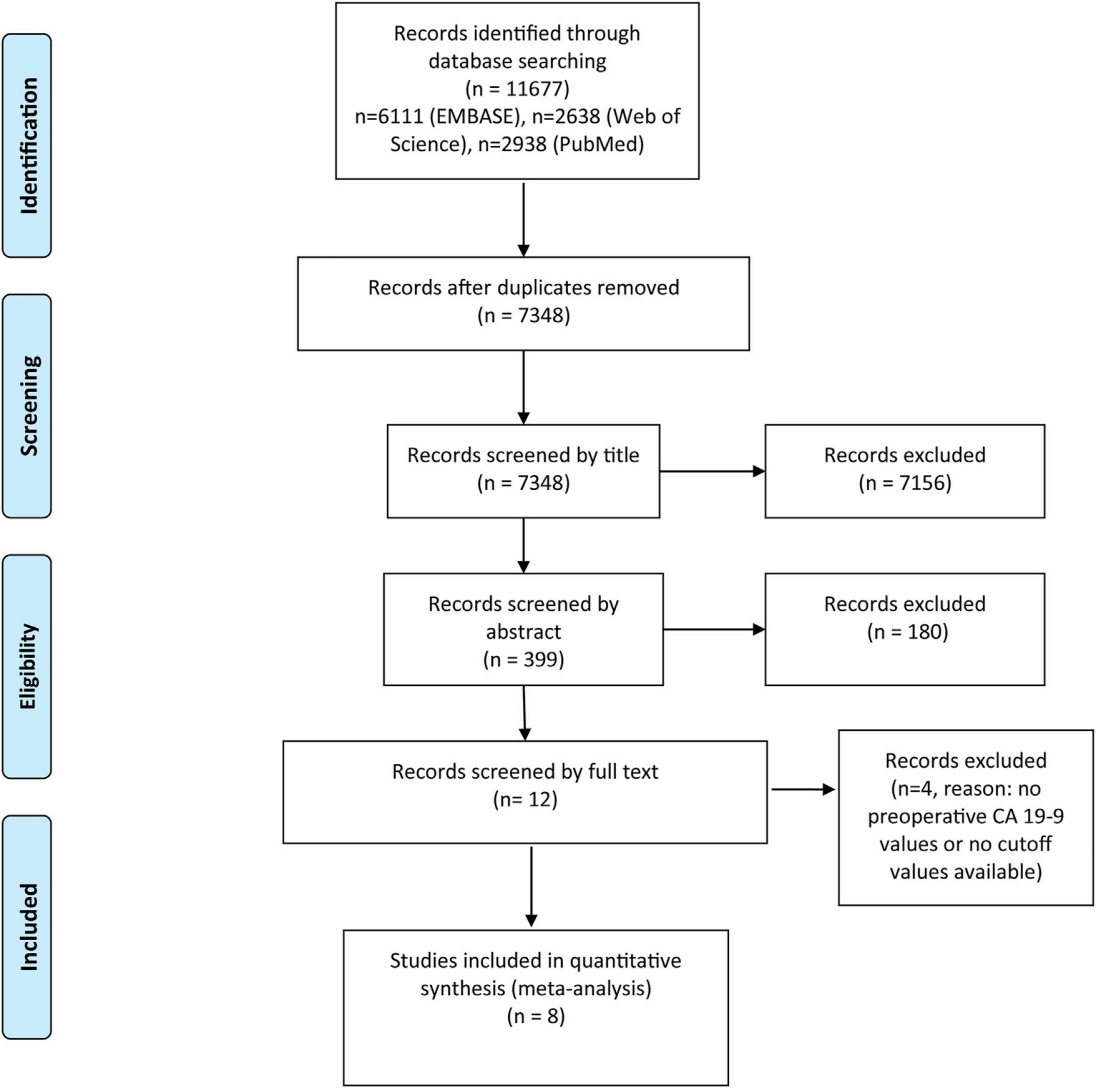
PRISMA flow diagram of included studies.

As a result of our systematic search, 11,677 records were found in total (EMBASE: 6111, PubMed: 2928, Web of Science 2638). The details of the selection process are shown in Figure 1. The remaining 12 articles were screened by full text; finally, eight articles were included in the quantitative synthesis [9–16].

### Value of Preoperative Serum CA 19-9 as a Predictor of Surgical Resectability

**FIGURE 2:**
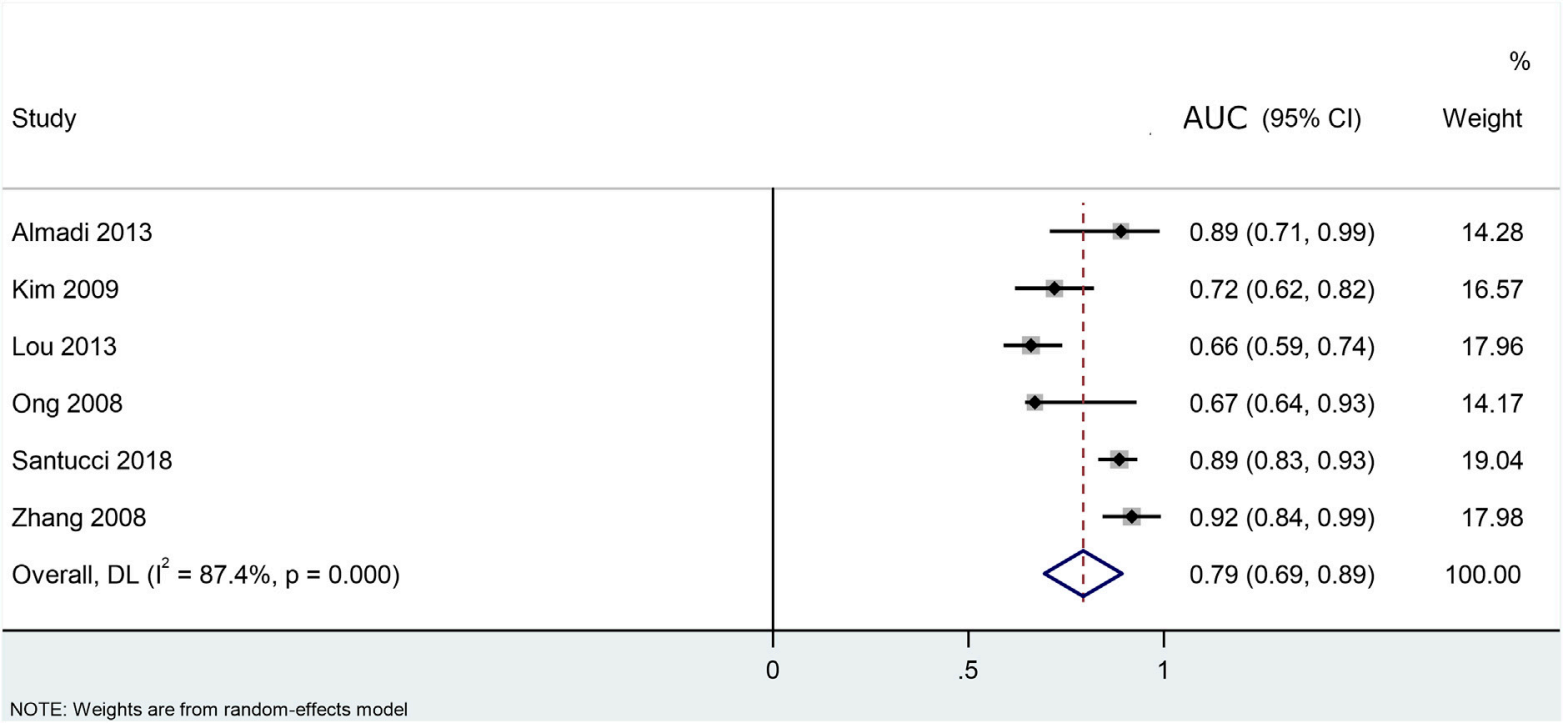
Area under the curves (AUC) describing the prognostic ability of CA 19- 9 to surgical resectability of pancreatic ductal adenocarcinoma.

**FIGURE 3:**
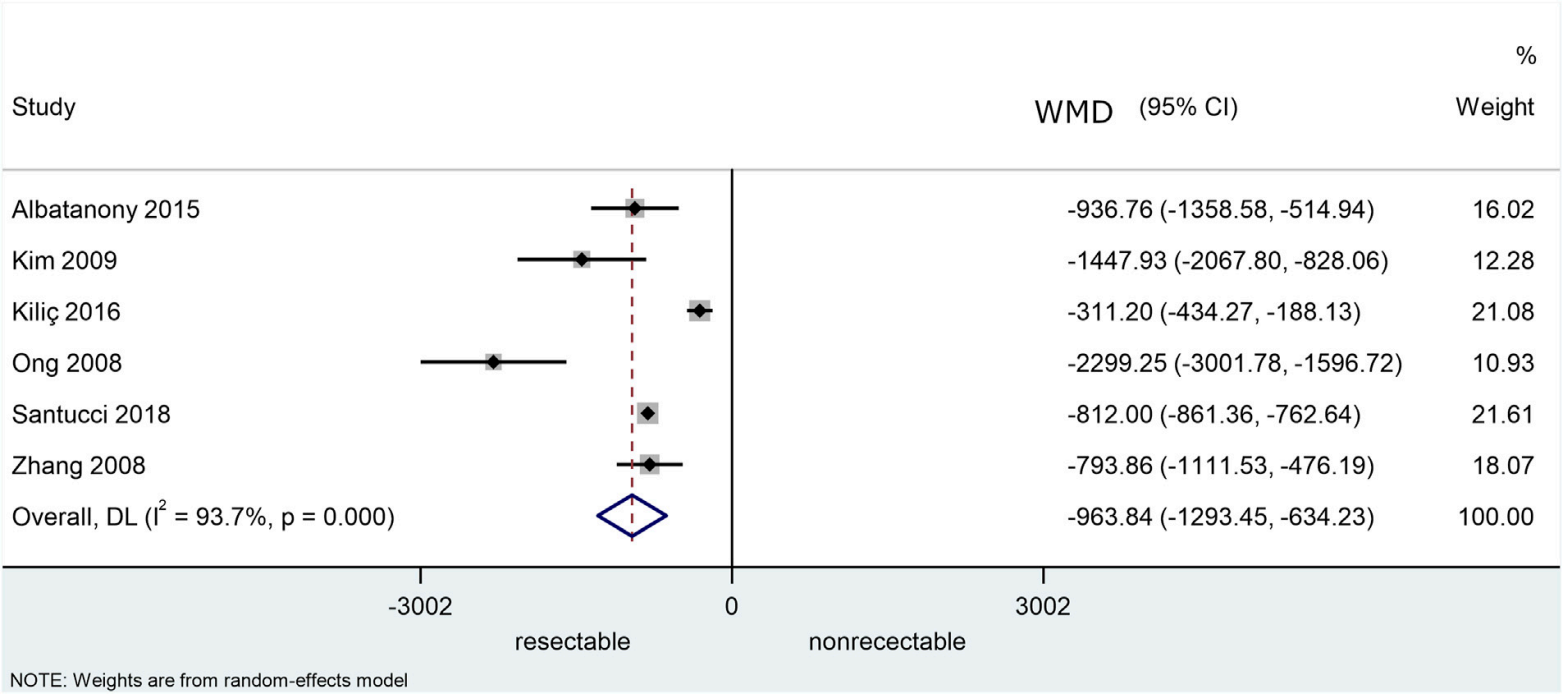
Weighted mean differences (WMD) in U/mL of the preoperative serum CA 19-9 levels between patients with resectable and unresectable ductal pancreatic adenocarcinoma.

Six out of the eight studies utilised receiver operating characteristic (ROC) curves in order to find the cut-off preoperative CA 19-9 value, above which the disease should be deemed unresectable. The overall result from the pooled AUC values from the ROC curves was 0.79 (CI: 0.694–0.893). Based on this, the preoperative CA 19-9 level is a “fair” marker of resectability but did not reach the 0.8 value, meaning that it is not classified as a “good” marker (Figure 2). The comparison of the preoperative CA 19-9 values showed a significant difference between the resectable and unresectable groups. The preoperative CA 19-9 values were 964 U/ml (*p* < 0.001) lower in the resectable group than in the unresectable group (Figure 3). However, based on the result of the I-squared test (87.4%, greater than 75%), considerable heterogeneity was present within the population. The pooled sensitivity and specificity values were calculated from the raw data of the studies (TP, TN, FP, and FN) (Figure 5).

### Risk of Bias Assessment Results

**FIGURE 4:**
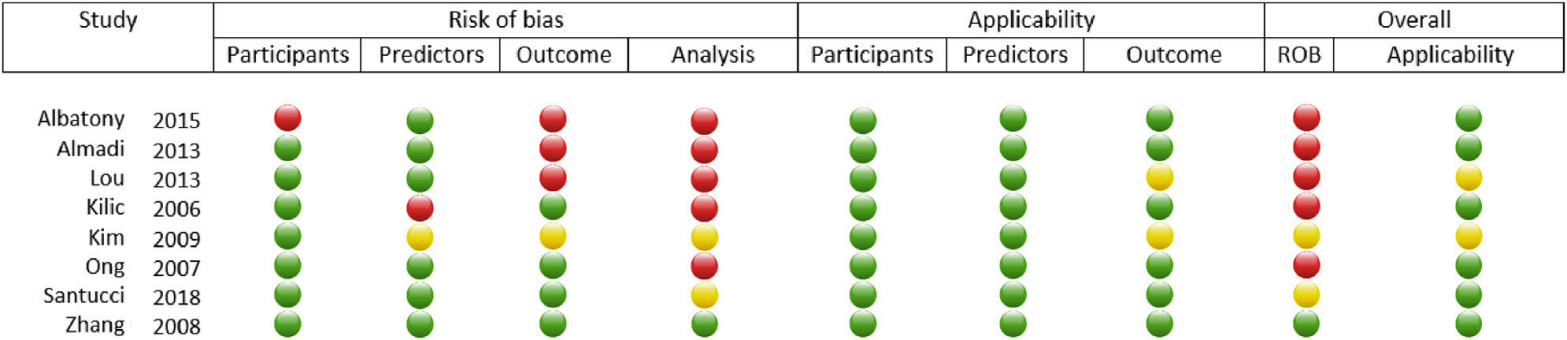
Visual representation of the results of the risk of bias assessment performed on the included articles using Cochrane’s PROBAST Tool. The green circles indicate low ROB or “yes” to applicability, yellow dots mean unclear ROB or applicability and red dots stand for high ROB or no applicability.

Regarding participants, almost all the studies had low risk of bias, except for one that had high risk of bias; all of them were of low concern of applicability (Figure 4). One study had high risk of bias regarding the participants, and one had unclear risk of bias; the rest of the studies had low risk of bias. All the studies had low concern of applicability for predictors. Three studies had high risk of bias for the outcome domain, and one had unclear risk of bias; the remaining three studies had low risk of bias for the outcome domain. All the studies had low concern for applicability regarding the outcome except for two studies that had unclear concern of applicability. In the analysis domain, four studies had high, two unclear and one study low risk of bias.

## Discussion

### Summary of Main Findings

Based these results, preoperative serum CA 19-9 is a fair marker of resectability of PDAC with a pooled AUC value of 0.79 (CI: 0.69–0.89). A meta-analytical calculation of the WMDs of the preoperative CA 19-9 levels was also performed between resectable and unresectable PDAC. The results showed that they are significantly higher in the case of the latter. Despite the significant difference, the usefulness of a fair marker is limited especially in clinical practice, so CA 19-9 on its own cannot be used for deciding resectability of PDAC.

### The Role of CA 19-9

CA 19-9 is the most widely researched and most specific tumor marker of pancreatic cancer known to date [2]. CA 19-9 has been examined as a prognostic perioperative biomarker predicting surgical resectability. In clinical practice, the frequent decision to make is how to start treating pancreatic cancer. It must be decided whether the patient should go through immediate surgery or chemotherapy. CA 19-9 as a prognostic marker for resectability would be a significant benefit because it is readily available from the blood with a serum test. There are previous reviews qualitatively summarizing its effectiveness in predicting resectability and survival rates, but until now, no meta-analysis has been published on this topic. In a systematic review, Poruk et al. described a close correlation between high preoperative CA 19-9 levels and R0 resectability as a result of their qualitative synthesis of the result [17]. In spite of the studies describing the usefulness of CA 19-9 predicting resectability, in clinical practice, CA 19-9 levels are not usually used on their own in determining the resectability of pancreatic cancer [18]. There is a clear positive correlation between the high CA 19-9 levels and the extent of the tumor mass and its spread in the body. It is widely used as monitoring the disease during chemotherapy or after surgery to detect its recurrence [19]. Our statistical analysis showed that the preoperative CA 19-9 levels were significantly higher in the unresectable cases. However, it was not possible to determine a specific cut-off value of resectability using the data available from the sources of this meta-analysis. In addition, in spite of the positive correlation between the higher CA 19-9 levels and unresectability, the heterogeneity within the population was too high, therefore it cannot be used as a marker of unresectability by itself. There are no widely used guidelines as of yet which include preoperative serum CA 19-9 in the evaluation of surgical resectability [20]. On the other hand, in the clinical practice, in cases of extremely high values of CA 19-9 levels, the disease is deemed unresectable; however, these should not be considered unresectable solely on preoperative CA 19-9 levels, because, as our results showed, it cannot serve as a single decisive factor. Further studies can incorporate CA 19- 9 level with other important parameters, such as imaging results and the general conditions of the patient, as well creating new scoring systems for the surgical resectability of PDAC. In the future, further markers could be assessed for predictive surgical potential. It would make surgical planning more effective and increase the rate of surgical resectability while lowering the intraoperatively discovered unresectable cases.

### Future Options for Dealing With Pancreatic Cancer

It can take up to 10 years for a tumor cell in the pancreas to cause the development of metastasis in other distant organs. Therefore, in theory, there is a large window of opportunity for the early detection of cancer [21, 22]. The ultimate goal would be to take this opportunity and find an early diagnostic biomarker in order to prevent patients from reaching the time when they are usually diagnosed. There are various biomarkers that could theoretically be used for diagnosing pancreatic cancer. CA 19-9 is unfortunately not suitable as an early screening biomarker of pancreatic cancer, because it has low positive predictive value due to the fact that there is a low number of patients with pancreatic cancer in the general population [23, 24]. Many new possible biomarkers are being studied and researched, such as markers on the surface of extra-cellular vesicles in human pancreatic juice [25]. Other potential novel serum markers have also been examined consisting of different protein markers, DNA methylation, cell-free nucleosomes, MicroRNAs, cell-free tumor DNA and multimarker panels [26]. However, they are not nearly as widely tested as CA 19-9, and they need to be tested on a larger clinical scale and over a longer time period in order for their effectiveness to be more certain [27]. To date, there is a lack of reliable, readily available early diagnostic modality for pancreatic cancer. More readily available and less invasive tests are also being researched in order to aid the early diagnosis in pancreatic cancer [28]. In the long term, early diagnosis is the main goal. As the research of the biology of this disease advances further and more reliable markers become available, earlier detection or even a population-wide screening will become available.

### Strengths

There are numerous strengths of this meta-analysis. The studies included in the analysis contain relevant evidence to the review question. The studies are eligible to address the objectives of this study. All the participants (the population described in the Methodology section), the interventions, the comparators and the outcomes were investigated. Eight studies which precisely fit our inclusion criteria have been included, and the overall number of participants across the studies was 848. The systematic review and meta-analysis were carried out following the predefined protocol, and achieved the aims stated in the introduction.

### Limitations

**FIGURE 5:**
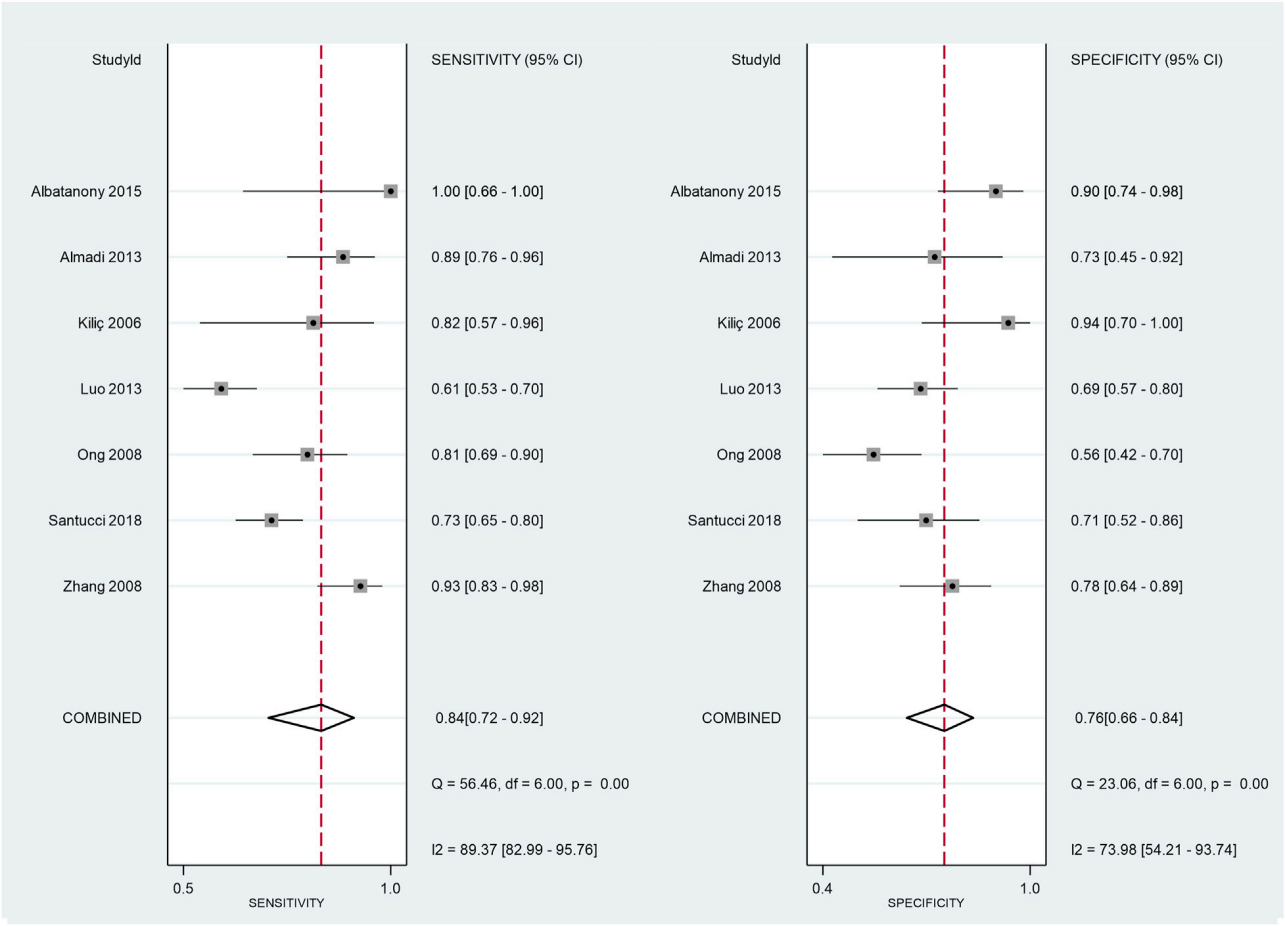
Pooled sensitivity and specificity values were calculated from the raw data of the studies [True Positive (TP), True Negative (TN), False Positive (FP), False Negative (FN)].

This presented meta-analysis also had several limitations that need to be discussed. Firstly, it is difficult, to define surgical resectability in a purely objective way, that fits all expectations. Some only regard those cases resectable that are R0 after the histological examination. There are others, who consider resectability in an intraoperative manner, so in their eyes not only R0 but also R1 and R2 fit the criteria. The results of the I- squared tests showed considerable (>75%) heterogeneity within the examined population in both cases. The most possible cause of this is the demographic difference, and another reason may be the differences within the same population due to the variations of the high variability in the course of the disease. The major inherent limitations of CA 19-9 are that it is a sialylated Lewis antigen, it is present on the surface of erythrocytes, 10% of the Caucasian population does not possess this antigen on the surface of the erythrocytes at all, and therefore, in their case, any measurement regarding CA 19-9 will be negative [29]. There are further obstacles in terms of routinely using CA 19-9 that pertain to its dynamics in certain medical conditions, such as obstructive jaundice. In case of these patient perioperative CA 19-9 levels could’ve been affected by high serum bilirubin levels, but this could be only solved by the high number of patients in the included studies. In the future patients with jaundice before the operation should make up a different subgroup to rule this factor out, however in this study there was no sufficient data available for subgroup analysis. CA 19-9 levels can be elevated in other gastrointestinal malignancies with hepatic, esophageal, or gastric origin [30]. Furthermore, its serum concentration can be elevated due to pancreatitis or cirrhosis. Biliary obstruction and the rise in serum bilirubin levels elevate serum CA 19-9 levels, making its application somewhat difficult to adjust [31]. An SROC analysis from the extracted data was planned to be performed; however, it was not possible as the number of the included studies with sufficient quality of data were too low for that [8]. Including more than the eight studies we have included in the final selection would have caused substantial decrease in the quality of the meta- analysis. These articles had the best available data to perform the statistical analysis, but was not sufficient to perform a reliable SROC analysis as described in the article written by Catherine M. Jones et al. [8] We have calculated the pooled sensitivity and specificity from the raw data extracted from the included studies. The results from this partially supported our conclusion (Figure 5). These values of pooled sensitivity and specificity are not sufficient to base therapeutical decisions on them in case of such a severe malignant disease. In addition, a subgroup analysis from the data was planned to further investigate the considerable heterogeneity of the data, but the available data was insufficient for that purpose. Another major limitation is that the authors of the articles included did not always publish the method they used for measuring CA 19-9; therefore, there could be discrepancies between the studies.

Although the overall pooled AUC result was 0.794 meaning that CA 19-9 is a “fair” marker statistically and might look encouraging, based on our data in the clinical decision making that single marker cannot be used because of the heterogenous nature of our patient population. However, the heterogeneity of data would have been corrected with more thorough inclusion criteria of the literature screening, the suitable methodologies as prospective clinical studies cannot be designed to answer this specific question without harming patients and be unethical. Taking together our pure data-based e findings and the nature of the disease in real clinical settings we feel that this pooled AUC value even though it is “fair”, it is not a “good” marker enough to be used decide against the surgical treatment of a patient solely using that. Based on our findings we feel the only conclusion we can draw up is CA19-9 cannot be used as a single marker, however patients above the cut-off have to be discussed more thoroughly taking more aspects with different weights into account. Refinement of these aspects requires further investigations.

There is a great difference in sensitivity, specificity and AUC values across the included studies.

The main reason for this is the heterogenous nature of this patient population. The other reason from studies is that there were slight differences in patient selection. Although this did not alter the patient inclusion or exclusion criteria of this meta-analysis. As discussed above theses reasons would have been covered up with planning of prospective randomised trials with more thorough inclusion criteria. Unfortunately, regarding the ethical statements for answering that question randomisation is not allowable. The statistical methods we used are attended to be suitable for smoothing out the heterogeneity but based on the previously discussed limitations they did not to prove the marker good enough to be suitable for a single parameter decision making.

## Conclusion

This study has provided the first objective evidence of that preoperative serum CA 19-9 levels cannot predict surgical resectability of pancreatic ductal adenocarcinoma alone. This does correlate with the clinical practice in that preopeperative examination should be very thorough in case of pancreatic ductal adenocarcinoma patients and unresectability shouldn’t be declared solely based on preoperative serum CA 19-9 levels.

As seen in our results, there is some form of correlation between the high preoperative serum CA 19-9 levels and the surgical inoperability of PDAC; however, it should not be used to determine unresectability on its own in everyday practice. Although CA 19-9 is not suitable alone in the surgical planning process, used together with other parameters, it could be a valuable candidate to create scoring systems to help decide the resectability of PDAC pre-operatively. Future studies could be used to establish a scoring system for resectability of pancreatic cancer incorporating preoperative CA 19-9 levels. This could also be used in difficult scenarios, such as deciding between primer surgery and neoadjuvant chemotherapy for borderline resectable pancreatic cancer.

## Supplementary Material

Supplementary Table S1A-C Characteristics of the included studies and the measured outcomes.

## Abbreviations

AUC, area under curve; CA 19-9, carbohydrate antigen 19-9; CT, computed tomography; ERCP, endoscopic retrograde cholangiography; EUS, endoscopic ultrasound; FN, false negative FP, false positive; MRI, magnetic resonance imaging; PDAC, pancreatic ductal adenocarcinoma; PET-CT, positron emission computed tomography; ROB, risk of bias; ROC, receiving operation characteristic TN- true negative; TP, true positive; WMD, weighed mean difference.
