# Supplementary material for: Preoperative Serum Carbohydrate Antigen 19-9 Levels Cannot Predict the Surgical Resectability of Pancreatic Cancer: A Meta-Analysis
Source: Pathol Oncol Res. 2022 May 10;28:1610266. doi: 10.3389/pore.2022.1610266 (PMC9136945; doi:10.3389/pore.2022.1610266)
Supplement: Supplementary file 1 [file Table1.pdf]

Table 1a: Characteristics of the included studies and the measured outcomes

First Author	Country	Age		Gender		Centers	Design	Type of study	Number of patients with preoperative CA 19-9 values available
		Resectable	Unresectable	Male	Female				
Albatanony 2015	Egypt	50,6 (+- 13,6 or 51-77)	59,4 (+- 11,4 or 48-76)	26	14	single	retrospective	cross sectional	40
Almadi 2013	Saudi Arabia	61,2 (+- 1,51)		36	25	single	retrospective	cross sectional	61
Luo 2013	China	60,7 (+-9,1)		115	97	single	retrospective	cross sectional	212
Kiliç 2006	Turkey	56 (38-74)		28	23	single	prospective	cross sectional	33
Kim 2009	South Korea	66,0 (+-10,2 or 41-88)		60	54	single	retrospective	cross sectional	114
Ong 2008	UK	63 (30-80)	66,5 (30-80)	64	45	single	retrospective	cross sectional	113
Santucci 2018	France	63,4 (+- 12,3)	64,6 (+- 13,4)	97	74	single	retrospective	cross sectional	171
Zhang 2008	China	59,0 (+-9 or 41-75)		72	32	single	retrospective	cross sectional	104
