# Supplementary material for: Preoperative Serum Carbohydrate Antigen 19-9 Levels Cannot Predict the Surgical Resectability of Pancreatic Cancer: A Meta-Analysis
Source: Pathol Oncol Res. 2022 May 10;28:1610266. doi: 10.3389/pore.2022.1610266 (PMC9136945; doi:10.3389/pore.2022.1610266)
Supplement: Supplementary file 2 [file Table2.pdf]

| Table 1b: Characteristics of the included studies and the measured outcomes | | | | | | | | | | | |
|---|---|---|---|---|---|---|---|---|---|---|---|
| First Author | Number of patients with resectable pancreatic cancer | Number of patients with unresectable pancreatic cancer | preoperative CA19-9 level cutoff value (U/mL) | Area Under the Receiving Operation Characteristics Curve (AUC) | 95% confidence interval (CI) | Sensitivity (%) | Specificity (%) | True Positives | False Positives | True Negatives | False Negatives |
| Albatanony 2015 | 30 | 10 | 307.55 | na | na | 100,00 | 88,67 | 9 | 3 | 28 | 0 |
| Almadi 2013 | 15 | 46 | 166,00 | 0,890 | 0,708-0,989 | 89,00 | 75,00 | 41 | 4 | 11 | 5 |
| Luo 2013 | 76 | 136 | 289.40 | 0,660 | 0,590–0,740 | 63,24 | 71,05 | 86 | 22 | 50 | 54 |
| Kiliç 2006 | 18 | 15 | 256,40 | 0,892 | na | 82,40 | 92,30 | 14 | 1 | 15 | 3 |
| Kim 2009 | 24 | 90 | 92,77 | 0,720 | 0,619-0,821 | 67,80 | 75,00 | na | na | na | na |
| Ong 2008 | 55 | 58 | 473,00 | 0,670 | 0,645-0,930 | 81,80 | 55,60 | 47 | 24 | 31 | 11 |
| Santucci 2018 | 49 | 122 | 178,00 | 0,886 | 0,832-0,932 | 81,90 | 81,60 | 102 | 9 | 22 | 38 |
| Zhang 2008 | 58 | 46 | 353,15 | 0,918 | 0,843-0,992 | 93,10 | 78,30 | 54 | 10 | 36 | 4 |
