# Supplementary material for: Preoperative Serum Carbohydrate Antigen 19-9 Levels Cannot Predict the Surgical Resectability of Pancreatic Cancer: A Meta-Analysis
Source: Pathol Oncol Res. 2022 May 10;28:1610266. doi: 10.3389/pore.2022.1610266 (PMC9136945; doi:10.3389/pore.2022.1610266)
Supplement: Supplementary file 3 [file Table3.pdf]

Table 1c: Characteristics of the included studies and the measured outcomes

First Author	Number of patients with resectable pancreatic cancer	Number of patients with unresectable pancreatic cancer	preoperative CA19-9 level cutoff value (U/mL)	Area Under the Receiving Operation Characteristics Curve (AUC)	95% confidence interval (CI)	Sensitivity (%)	Specificity (%)	Positive predictive Value (%)	Negative Predictive Value (%)	True Positives	False Positives	True Negatives	False Negatives
Albatanony 2015	30	10	307.55	na	na	100,00	88,67	71,43	100,00	9	3	28	0
Almadi 2013	15	46	166,00	0,890	0,708-0,989	89,00	75,00	91,61	56,08	41	4	11	5
Luo 2013	76	136	289.40	0,660	0,590–0,740	63,24	71,05	79,63	51,92	86	22	50	54
Kiliç 2006	18	15	256,40	0,892	na	82,40	92,30	91,40	83,90	14	1	15	3
Kim 2009	24	90	92,77	0,720	0,619-0,821	67,80	75,00	na	na	na	na	na	na
Ong 2008	55	58	473,00	0,670	0,645-0,930	81,80	55,60	66,01	0,74	47	24	31	11
Santucci 2018	49	122	178,00	0,886	0,832-0,932	81,90	81,60	92,20	70,00	102	9	22	38
Zhang 2008	58	46	353,15	0,918	0,843-0,992	93,10	78,30	84,38	90,00	54	10	36	4
